# What’s Age Have to Do With Comfortableness in Seeking and Providing Social Support After a Disaster?

**DOI:** 10.1093/geroni/igaa057.1422

**Published:** 2020-12-16

**Authors:** Judith Robertson Phillips, Edith Jimenez

**Affiliations:** California State University San Marcos, San Marcos, California, United States

## Abstract

Disasters are associated with loss of property and loss of psychological well-being. Receiving various types of social support, such as emotional or tangible support, from various sources, such as family or friends, have been found to reduce the adverse impact of a disaster on affected adult community residents. It is not well studied, though, why some adults will not seek or provide support after a disaster while other adults will. The purpose of this study was to explore how age might play a role in how comfortable adults were in seeking social support from others and how age might play a role in how comfortable adults were in providing social support to others after a disaster, the 2014 San Diego County, CA wildfires. One hundred and twenty-two community residents (18 to 80 years) were recruited with 33 adults identified as Secondary Disaster Survivors and 89 adults identified as Non-Victims. Analyses revealed that age did play a role in Secondary Survivors’ comfortableness in seeking support from others; Older Adults (50-80) were significantly less likely than Middle-aged Adults (30-49) to feel comfortable in seeking support. There was no significant comfortableness difference between Middle-aged and Younger Adults (18-29) in seeking support. There were no age group differences in comfortableness in providing support to others for Secondary Survivors and Non-Victims. Implications from this data suggest that local disaster organizations should actively support affected older adults post-disaster while also including unaffected adults of all ages in the process of providing support to those in need.

